# Microstructure Analysis of Ti-xPt Alloys and the Effect of Pt Content on the Mechanical Properties and Corrosion Behavior of Ti Alloys

**DOI:** 10.3390/ma7053990

**Published:** 2014-05-21

**Authors:** Ho-Jun Song, Mi-Kyung Han, Hyeon-Gyeong Jeong, Yong-Tai Lee, Yeong-Joon Park

**Affiliations:** 1 Department of Dental Materials and MRC for Biomineralization Disorders, School of Dentistry, Chonnam National University, Gwangju 500-757, Korea; E-Mails: songhj@jnu.ac.kr (H.-J.S.); mikihan@jun.ac.kr (M.-K.H.); moguree@nate.com (H.-G.J.); 2 Korea Institute of Materials Science, Changwon 642-831, Korea; E-Mail: ytlee@kims.re.kr

**Keywords:** Ti-Pt alloys, corrosion resistance, microstructure, mechanical properties

## Abstract

The microstructure, mechanical properties, and corrosion behavior of binary Ti-xPt alloys containing 5, 10, 15 and 20 wt% Pt were investigated in order to develop new Ti-based dental materials possessing superior properties than those of commercially pure titanium (cp-Ti). All of the Ti-xPt (x = 5, 10, 15, 20) alloys showed hexagonal α-Ti structure with cubic Ti_3_Pt intermetallic phase. The mechanical properties and corrosion behavior of Ti-xPt alloys were sensitive to the Pt content. The addition of Pt contributed to hardening of cp-Ti and to improving its oxidation resistance. Electrochemical results showed that the Ti-xPt alloys exhibited superior corrosion resistance than that of cp-Ti.

## 1. Introduction

Ti-based alloys have received widespread attention due to their favorable mechanical properties including high specific strength, good corrosion stability, and biocompatibility after implantation [1,2]. However, cp-Ti has several inherent problems such as low deformability, low wear resistance, and difficulty in manufacturing, welding and machining [3,4]. The other drawbacks of Ti are a high melting point (~1700 °C) and a high reactivity with surrounding impurities such as oxygen and nitrogen at elevated temperatures [5]. Therefore, titanium is still unsatisfactory for practical applications. Attempts have been made to develop new titanium alloys that have improved mechanical properties and castability by alloying Ti with a variety of elements. Although many types of titanium alloys are available, Ti-6Al-4V alloy is the most widely used Ti alloy. However, the release of Al and V ions from the alloy causes cytotoxic effects and neurological disorders [6]. Therefore, non toxic elements, such as Nb, Pt, Pd, and Ta, can be the new candidates for developing new Ti-based implant materials.

Recently, Ti-Pt binary alloys have been developed, and these alloys were expected to become promising candidates for dental applications due to alloying with non-toxic elements, better grindability, and better mechanical compatibility with bone tissue than cp-Ti and Ti-6Al-4V alloys. The addition of a small amount of Pt or Pd to Ti was very effective in improving the corrosion resistance of titanium due to promotion of the active-passive transition by the enhanced cathodic reaction [7]. It has been known that alloying Ti with noble metals such as, Pt, Pd, Re, Ru, Ir, Os, Rh, and Au, markedly improves its corrosion resistance to H_2_SO_4_, HC1, and other hydrogen-saturated acid media [8]. The amount of Pt released from the Ti alloys containing 0.5 wt% Pt was considerably less than that released from pure Ti or Ti alloys without Pt [9]. However, very few papers have demonstrated the effect of noble Pt metal alloying on the mechanical properties of titanium.

In the present study, with the goal of developing a dental titanium alloy with better mechanical properties than cp-Ti, the effect of alloying element Pt on the microstructure, mechanical properties, and corrosion behavior of Ti-Pt binary alloys, with the addition of 5, 10, 15 and 20 wt% Pt, was investigated.

## 2. Results and Discussion

### 2.1. Phase and Microstructure

**Figure 1.**
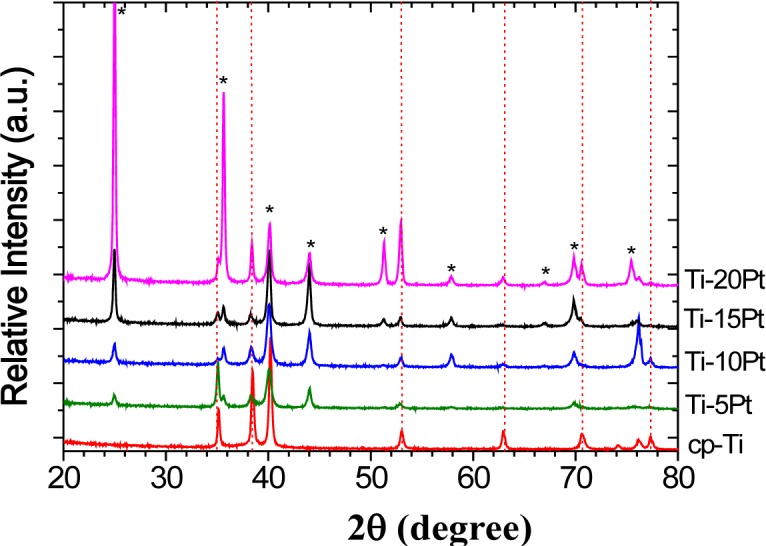
XRD patterns of cast cp-Ti and series of binary Ti-xPt alloys. The vertical dotted line in the figure is a guide to the eye, and it connects the peak positions of the α-Ti phase. Diffraction peaks corresponding to cubic Ti_3_Pt are marked with a star shape (*). A gradual increase in diffraction peak intensities for cubic phase is observed as a function of increased Pt content.

X-ray diffraction (XRD) analysis was conducted for characterization of the phase of the cast alloy. The phases were identified by matching each characteristic peak with the JCPDS files (JCPDS card No.44-1294 for α-Ti and JCPDS card No.00-018-0979 for Ti_3_Pt) [10]. The XRD patterns of cast cp-Ti and series of binary Ti-xPt alloys are shown in Figure 1. The XRD peaks of binary Ti-xPt alloys were comprised predominantly of Ti_3_Pt and α-Ti, which were in agreement with those in the reported phase diagram [11,12]. According to the Ti-Pt phase diagram, the formation of Ti_3_Pt can be predicted by the eutectoid reaction (β-Ti → α-Ti + Ti_3_Pt) at a Pt concentration of 12.0 wt% [13]. In the alloys examined in the present investigation, Ti_3_Pt was formed by the addition of a small amount of Pt to Ti. The amount of Ti_3_Pt phase increased linearly as the content of Pt was increased.

**Figure 2.**
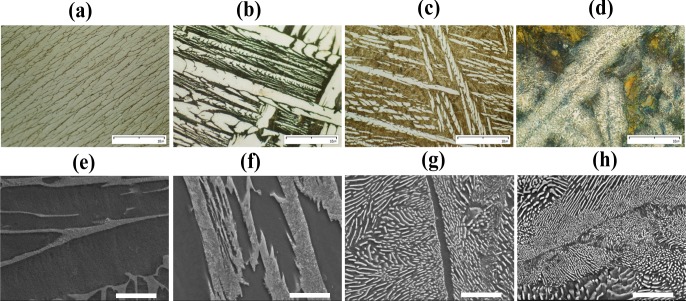
Optical micrographs (×400) of Ti-xPt alloys: (**a**) Ti-5Pt; (**b**) Ti-10Pt; (**c**) Ti-15Pt; (**d**) Ti-20Pt; and SEM micrographs of Ti-xPt alloys using secondary electron detector: (**e**) Ti-5Pt; (**f**) Ti-10Pt; (**g**) Ti-15Pt; (**h**) Ti-20Pt. Scale bar = 10 μm.

Microstructure observation of Ti-xPt alloys with different Pt contents (5, 10, 15, and 20 wt%) was performed by optical microscopy and SEM with the secondary electron detector, as shown in Figure 2. Microstructural examination showed that significant amounts of second phase were observed in all of the samples, and this was in agreement with phase identification by XRD. With the aid of energy dispersive x-ray (EDX) analysis, the light region in optical micrographs corresponded to the Ti matrix and the dark region corresponded to a high Pt-content phase as shown in Figure 2a–d, whereas the high Pt-content phase was observed as a light contrast in SEM of polished surfaces, as shown in Figure 2e–h. The EDX showed that the atomic ratio of Ti and Pt of the intermetallic phase was 3:1. It was clearly observed that the amount of Ti_3_Pt phase increased with increasing Pt content in the sample.

**Figure 3.**
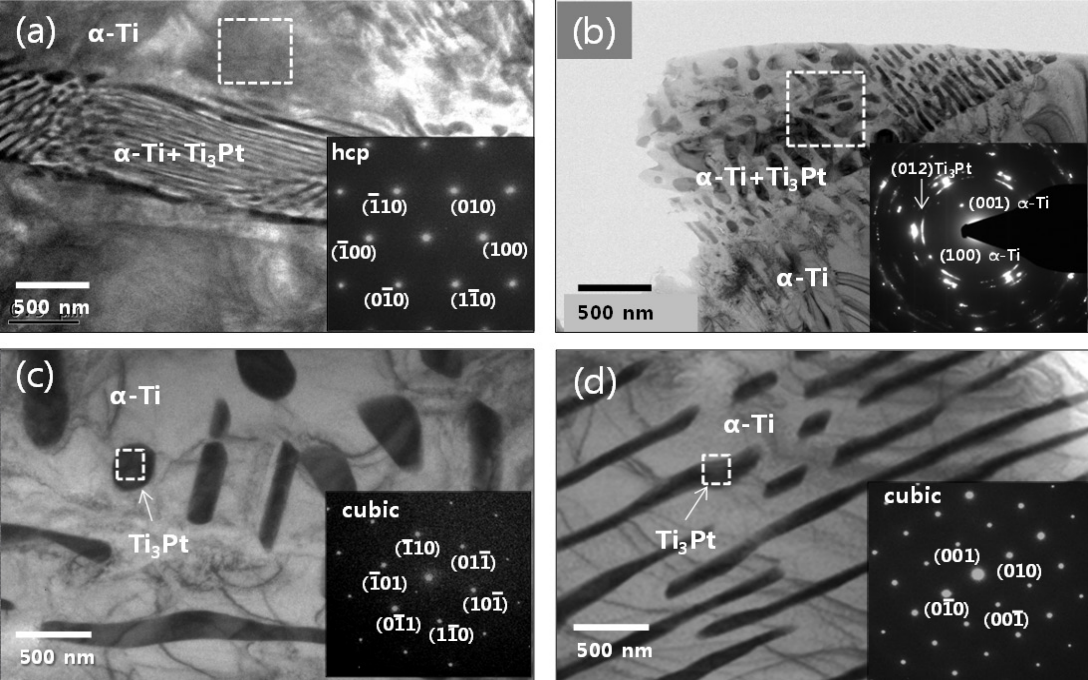
TEM images and selected area energy diffraction (SAED) patterns (inset) of eutectoids of Ti-xPt alloys: (**a**) Ti-5Pt; (**b**) Ti-10Pt; (**c**) Ti-15Pt; (**d**) Ti-20Pt.

The typical TEM images of Ti-xPt alloys along with SAED patterns obtained from the marked region in each TEM image are shown in Figure 3. In all of the investigated Ti-xPt (x = 5, 10, 15, and 20 wt%) alloys, the microstructure appeared as a fine eutectic lamellar structure consisting of α-Ti and Ti_3_Pt phases. The matrix was composed of α-Ti phase as indicated by the SAED pattern in Figure 3a. The dark region corresponded to the Ti_3_Pt phase. The corresponding SAED patterns of dark regions in the TEM image could be indexed in terms of (111) zone axis (inset in Figure 3c) and (100) zone axis (inset in Figure 3d) of a cubic structure of Ti_3_Pt with the lattice parameter *a* = 5.0327 Å, which was found to be in good agreement with the reported value of Ti_3_Pt [14]. It is worth mentioning that the Ti_3_Pt phase was precipitated as the nanostructure in alloys with a low Pt content, as shown in Figure 3a,b, and hence the precipitated Ti_3_Pt phase became the microstructure with a high Pt content, as shown in Figure 3c,d.

**Figure 4.**
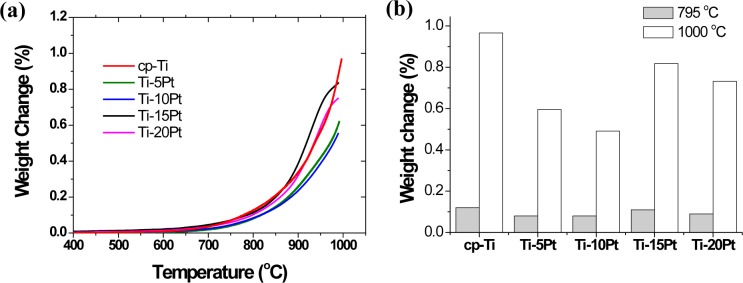
Thermogravimetric analysis (TGA) of cp-Ti and Ti-xPt alloys showing various degrees of weight gain (%) after heating in air. (**a**) TGA curves showing a single parabolic increment in mass; (**b**) various degrees of weight gain (relative wt%) by heating in air up to 795 °C and 1000 °C.

The oxidation behavior of Ti-xPt alloys was assessed using thermogravimetric analysis (TGA). Figure 4 shows the result of the TGA experiment when the cp-Ti and Ti-xPt alloys were heated up to 795 and 1000 °C at a heating rate of 10 °C/min in air. Each of the samples was oxidized, and the weight gain in the oxidized samples was compared to the weight gain in non-oxidized samples. All of the samples showed a single parabolic increment in mass during oxidation, shown in Figure 4a. Between room temperature to 580 °C, the change in mass was not observed in all of the Ti-xPt alloys, which was indicative of the oxidation resistance. At a temperature higher than 580 °C, the Ti-xPt alloys were rapidly oxidized, resulting in a significant increase in mass. The weight change was increased by increasing the oxidation temperature. All of the Pt-containing samples showed weight gain of about 0.1%–0.2% at a temperature of 795 °C, whereas at 1000 °C, a significant change in mass of 0.6%–0.8% was observed. The final mass change in the Ti-xPt alloys was significantly less than that after the oxidation of cp-Ti, indicating that the addition of Pt to cp-Ti could restrain the oxidation rate of the alloy, and Ti-xPt alloys had higher oxidation protection ability. The weight gain was decreased by increasing the Pt content up to 10 wt%, and then it was increased by increasing the Pt content further.

### 2.2. Mechanical Properties

The mechanical properties of titanium alloys were influenced by the fine microstructure of the alloys, chemical composition, and volume fraction of the constituent phases. We investigated the effect of addition of alloying element Pt on the mechanical properties and corrosion behavior of Ti. Table 1 shows Vickers hardness and elastic modulus values for all of the Ti-xPt alloys in comparison with those of cp-Ti. All of the Ti-xPt alloys had higher Vickers hardness values (VHN) (*p* < 0.05) than that of the cp-Ti (165 VHN). This result indicated that the Pt element can effectively increase the microhardness values of the Ti. This increase in hardness was probably caused by the formation of a eutectoid structure consisting of α-Ti and the intermetallic Ti_3_Pt compound. A similar enhancement in hardness due to the precipitation of the intermetallic compound was observed in the Ti-7Cr system [15] and the Ti-30Nb system [16]. It has been known that the hardness is also affected by the grain size of the precipitates. It is reported that the hardness of polycrystalline materials decreases with increasing grain size [17]. The Ti-10Pt alloy exhibited the highest hardness value of 603 VHN due to the precipitation of Ti_3_Pt as “nanophases”. With a further increase of Pt content in Ti alloys, the microhardness decreased, which can be attributed to the formation of Ti_3_Pt as “microphases”. Therefore, among the Ti-xPt alloys, Ti-20Pt showed the lowest hardness value. The elastic modulus values for Ti-xPt (x = 5, 10, 15, and 20) alloys were in the range of 139–147 GPa. These values were higher (*p* < 0.05) than that of the cp-Ti (132 GPa) due to the presence of a small fraction of the intermetallic Ti_3_Pt phase.

### 2.3. Corrosion Behavior

**Figure 5.**
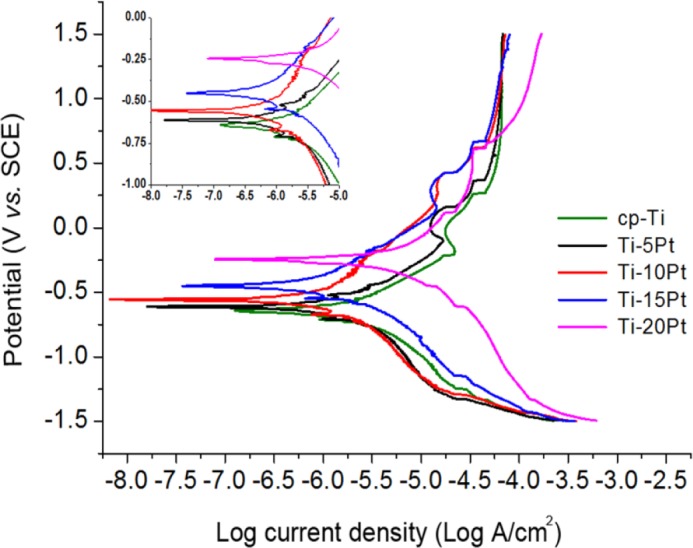
Representative potentiodynamic polarization curves for cp-Ti and Ti-xPt alloys. Inset shows enlarged polarization curve segments at the corrosion potential.

The corrosion behavior of Ti-xPt alloys was evaluated using potentiodynamic polarization and galvanic couple technique in order to investigate the effect of Pt content on the polarization curve to ascertain its suitability for dental implant applications. Potentiodynamic polarization curves of cp-Ti and Ti-xPt alloys were recorded at a sweep rate of 0.005 V/s, between the potential range of −1.5 and +1.5 V in NaCl solution, and the results are shown in Figure 5. All of the curves exhibited a shift of the electrochemical potential compared to that of cp-Ti. This shift indicated the positive influence of Pt on the corrosion resistance of cp-Ti. This is attributed to the promotion of the active-passive transition by the enhanced cathodic reaction [18]. Because the electronegativity of Ti (1.54) is lower than that of Pt (2.28), electrons move toward the region of Pt atoms in the alloy. The addition of Pt promotes oxygen reduction at the cathodic area around the Pt atoms, and thus the enhanced cathodic reaction accelerates spontaneous passivation of the Ti surface at the anodic area.

Using the Tafel extrapolation method, the corrosion potential (E_corr_) and corrosion current density (I_corr_) of cp-Ti and Ti-xPt alloys were calculated using both the anodic and cathodic branches of the potentiodynamic polarization curves, and they are listed in Table 2. Average corrosion potential of all the investigated Ti-xPt alloys was higher than that of cp-Ti. In comparison with cp-Ti, all of the investigated samples exhibited significantly reduced corrosion current density values. It was observed that the I_corr_ values decreased and the E_corr_ values increased towards positive direction for the Ti-xPt alloys, thus demonstrating an increased corrosion resistance of Ti-xPt than cp-Ti. The increase in corrosion resistance of Ti-xPt alloys was probably caused by the decrease of α-Ti phase by the formation of intermetallic Ti_3_Pt compound. There are reports of increased corrosion resistance of titanium alloys due to the addition of Pt element [8,9,18,19].

**Figure 6.**
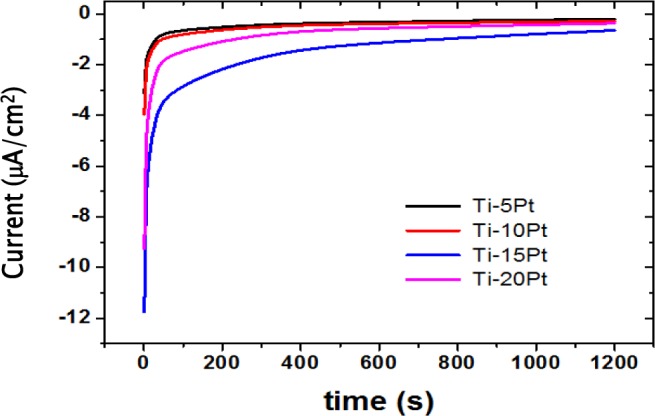
Mean values of galvanic currents *versus* time of couplings of cp-Ti/Ti-xPt alloys.

Passivation phenomenon may be better studied by the galvanic couple technique. Mean values of galvanic currents *versus* time of the couplings of cp-Ti/Ti-xPt alloys are shown in Figure 6. Current values initially exhibited a rapid increase and then a slow rise to attain a steady state current value. This general behavior might be explained by a reduction in the active area due to the growth of a passive film on Ti-xPt alloys. A steady state current value was attained more slowly for Ti-20Pt, indicating that a passive film grew more slowly on the Ti-20Pt alloy. The Ti-5Pt alloy showed the fastest tendency among the Ti-xPt alloys indicating that the formed film was more stable. This result might be mainly related to the coexistence of α-Ti phases and a small fraction of the intermetallic Ti_3_Pt phase.

## 3. Experimental Section

### 3.1. Material Preparation

Experimental Ti-Pt alloys (5, 10, 15, and 20 wt% Pt) were prepared by arc-melting the stoichiometric quantities of the elements on a water-cooled copper hearth using a tungsten electrode under a high-purity argon atmosphere. The starting materials (Ti sponge, Alfa Aesar, Ward Hill, MA, USA, 99.9%; Pt ingot, LS-Nikko, Kyunggi, Korea, 99.95%) were used without further purification. During the arc-melting procedure, a titanium getter was heated prior to melting the reactant mixture to further purify the argon atmosphere. The samples were remelted several times to promote sample homogeneity. Subsequently, the samples were heat treated using a tube furnace under argon atmosphere for 4 h at temperatures below 150 °C to the respective solidus temperatures followed by cooling down to 600 °C in a furnace at a rate of 10 °C/min and air-cooling to room temperature. These heat treatment conditions were chosen in accordance with the binary Ti-Pt phase diagrams [11,12]. Samples embedded in epoxy resin were cut and polished into disks of about 1.2 mm thickness with successively finer SiC papers up to #2000, and then ultrasonically cleaned in distilled water. Subsequently, the polished samples were etched with Keller’s solution (distilled water: 65% HNO_3_: 32% HCl: 40% HF = 95: 2.5: 1.5: 0.5).

### 3.2. Material Characterization

Phase analysis and structural characterization were performed by X-ray diffraction. The XRD diffraction patterns were collected for the bulk sample using a X’Pert PRO Multi Purpose X-Ray Diffractometer (40 kV and 40 mA) with Cu Kα (λ = 1.54056 Å). The scanning speed was 2°/min and the scanning angle ranged from 20° to 80° 2θ. The cp-Ti was used as a control. The lattice parameters were obtained by least squares refinement of data in the 20 range of 20°–80° with the aid of a Rietveld refinement program [20]. The microstructure of samples was examined using a metallurgical microscope (Epiphot FX-35WA, Nikon, Tokyo, Japan), scanning electron microscope (SEM; Hitachi, S-3000N, Tokyo, Japan), high-resolution transmission electron microscopy (HRTEM; Philips, Technai-F20, Eindhoven, Netherlands), selected area energy diffraction (SAED), and energy dispersive X-ray analysis (EDX; EMAX, Horiba, Tokyo, Japan). The phase transformation of Ti-xPt alloys was investigated by heating approximately 200 mg of the sample to 1000 °C at a rate of 20 °C/min using differential scanning calorimetry (DSC, DSC 404 C, Netzsch, Selb, Germany). The oxidation behavior of Ti with different Pt content was tested with TGA (Thermogravimetric Analysis, SDTA 851e, Mettler-Toledo Inc, Hightstown, NJ, USA), which measured the change in mass due to oxidation. The samples measuring 4.5 × 4.2 × 14.0 mm^3^ in size were heated to 1000 °C at a heating rate of 10 °C/min with an air flowing rate of 50 mL/min.

### 3.3. Measurement of Mechanical Properties

The microhardness of polished alloys was measured using a Vickers microhardness tester (Zwick, Postfach4350, Ulm, Germany) with a load of 500 g for 30 s. Elastic modulus measurement was performed using Nanoindenter XP (MTS Co, Eden Prairie, MN, USA) in a continuous stiffness measurement mode with the Berkovich type indenter. The indentations were made using a constant nominal strain rate of 0.05/s. The maximum indentation depth was 2 μm. A Poisson’s ratio of 0.35 was used to calculate the elastic modulus.

### 3.4. Electrochemical Analysis

Potentiodynamic anodic polarization test was conducted at a scan rate of 5 mV/s from −1.5 V to +1.5 V (*vs.* saturated calomel electrode, SCE) using a potentiostat (WAT 100, WonA Tech Co., Ltd, Seoul, Korea) in 0.9% NaCl solution at 37 ± 1 °C. At least three samples were tested to confirm the repetition of the experimental results. The surface of the sample with a 10 mm diameter was mechanically polished with SiC paper up to 2000 grit, and cleaned in an ultrasonic cleaner for 5 min. Potentiodynamic anodic polarization measurements were carried out after an immersion period of 1 h at the open circuit potential. The electrochemical measurements were recorded using the three electrode technique consisting of the working electrode (test samples), the counter electrode (high density carbon), and the reference electrode (saturated calomel electrode). Ar gas was bubbled through the electrolyte at 150 mL/min for more than 20 min to eliminate the residual oxygen in the electrolyte. The used electrolyte was replaced with a fresh electrolyte before each measurement. The exposed surface area of samples in the electrolyte was 0.283 cm^2^. The potentiodynamic polarization curves were plotted using an automatic data acquisition system. Both corrosion potential and current density were estimated by Tafel plots using both anodic and cathodic branches.

The galvanic voltage and the galvanic current density of various Ti-xPt/cp-Ti galvanic pairs were measured over a 20 min period by using Potentiostat/Galvanostat at ambient conditions (ZIVE SP2, WonA Tech Co., Ltd). The experimental setup for electrochemical measurements consisted of a three-electrode cell with the sample as a working electrode with an exposed area of 0.785 cm^2^, a SCE as a reference electrode, and cp-Ti as the counter electrode.

### 3.5. Statistical Analysis

Version 19.0 of the statistical software, SPSS (SPSS, Inc., Chicago, IL, USA), was used to analyze the data by means of the Kruskal–Wallis one-way analysis of variance and Duncan’s multiple range test with α = 0.05. Data were expressed as the mean ± standard deviation (SD) for each of the tests.

## 4. Conclusions

Experimental results indicated that both the microstructure and mechanical properties of the cast Ti-xPt alloys were sensitive to their Pt content. Based on the XRD and optical microscopy results, all of the Ti-xPt alloys had a hexagonal α-Ti phase with precipitation of cubic Ti_3_Pt intermetallic phases. By alloying Ti with Pt, the cast Ti-xPt alloys exhibited higher hardness and better oxidation protection ability. These enhanced mechanical properties of Ti-xPt alloys could have resulted from the formation of intermetallic Ti_3_Pt phase in Ti. It was found that the Ti-xPt showed better corrosion resistance than the cp-Ti. After considering the mechanical properties and corrosion behavior of all Ti-xPt alloys, Ti alloys with 5 wt% and 10 wt% Pt were found to be good candidates for dental casting alloys.

## Figures and Tables

**Table 1.** Vickers hardness and elastic modulus values of Ti-xPt alloys compared with cp-Ti ( number of measurements = 5).

Alloy Code	Vickers Hardness (VHN)	Elastic Modulus (GPa)
cp-Ti	164.54 ± 3.54 ^a^,^*^	132.35 ± 12.22 ^a^,^*^
Ti-5Pt	464.00 ± 4.00 ^d^	139.23 ± 4.45 ^a,b^
Ti-10Pt	603.00 ± 7.65 ^e^	146.25 ± 4.36 ^b^
Ti-15Pt	410.60 ± 11.22 ^c^	146.97 ± 5.11 ^b^
Ti-20Pt	387.60 ± 9.81 ^b^	143.60 ± 3.24 ^b^

* Within the same column, mean values with the same superscript alphabet were not statistically different at 5% (*p* > 0.05).

**Table 2.** Corrosion potential (E_corr_) and corrosion current density (I_corr_) of cp-Ti and Ti-xPt alloys (number of measurements = 3).

Alloy Code	E_corr_ (mV)	I_corr_ (logA/cm^2^)
cp-Ti	−550.30 ± 43.90 ^a^,^*^	−7.10 ± 0.07 ^b^,^*^
Ti-5Pt	−530.09 ± 83.25 ^a^	−7.16 ± 0.13 ^b^
Ti-10Pt	−509.60 ± 84.49 ^a^	−7.34 ± 0.22 ^a,b^
Ti-15Pt	−442.43 ± 115.77 ^a^	−7.47 ± 0.11 ^a^
Ti-20Pt	−410.97 ± 174.89 ^a^	−7.17 ± 0.02 ^b^

*Within the same column, mean values with the same superscript alphabet were not statistically different at 5% (*p* > 0.05).
